# A Methodology for Optimizing the Calibration and Validation of Reactive Transport Models for Cement-Based Materials

**DOI:** 10.3390/ma15165590

**Published:** 2022-08-15

**Authors:** Mouadh Addassi, Victor Marcos-Meson, Wolfgang Kunther, Hussein Hoteit, Alexander Michel

**Affiliations:** 1 Department of Civil Engineering, Technical University of Denmark, 2800 Kongens Lyngby, Denmark; 2 Physical Science and Engineering Division, King Abdullah University of Science and Technology, Thuwal 23955-6900, Saudi Arabia; 3 COWI A/S, 2800 Kongens Lyngby, Denmark

**Keywords:** reactive transport, cement, carbonation, experimental validation

## Abstract

Reactive transport models are useful tools in the development of cement-based materials. The output of cement-related reactive transport models is primarily regarded as qualitative and not quantitative, mainly due to limited or missing experimental validation. This paper presents an approach to optimize the calibration process of reactive transport models for cement-based materials, using the results of several short-term experiments. A quantitative comparison of changes in the hydrate phases (measured using TGA and XRD) and exposure solution (measured using ICP-OES) was used to (1) establish a representative chemical model, limiting the number of hydrate phases and dissolved species, and (2) calibrate the transport processes by only modeling the initial tortuosity. A case study comprising the early age carbonation of cement is presented to demonstrate the approach. The results demonstrate that the inclusion of a microstructure model in our framework minimizes the impact of the initial tortuosity factor as a fitting parameter for the transport processes. The proposed approach increases the accuracy of reactive transport models and, thus, allowing for more realistic modeling of long-term exposure.

## 1. Introduction

The deterioration of cement-based materials, such as carbonation, chloride ingress, sulphate attack, etc., remains a major societal challenge, despite many years of practical experience and a large number of extensive research programs. Although several challenges remain [1], the development of more advanced modeling tools has contributed to an increased understanding of the underlying mechanisms and processes related to the deterioration of cement-based materials. Noteworthy is the development of reactive transport (RT) models to simulate changes in cement-based materials due to transport and chemical interaction between the initial material and ionic or gaseous species from the surrounding environment [2]. The application of RT models is gaining interest in the cement and concrete community [3,4,5,6,7,8,9], a process driven by continued development and improved capabilities of RT model [6,8,10,11,12,13,14], as well as the development of thermodynamic databases for cement-based materials, [15,16,17,18,19].

While these models help to shed light on some of the governing deterioration mechanisms of cement-based materials, these tools require a large number of input parameters as well as extensive experimental data for model testing and validation. This is due to the complexity of cement systems, where a large number of chemical species can be considered to conceptualize and study the mechanisms of interest. This high degree of freedom in the input design space is challenging for validating and testing RT models for cement-based systems. Typically, experimental studies are only suitable for limited validation and testing of RT modeling tools, such as mass transport of selected species [20,21,22,23]. The most common practice when evaluating reactive transport models is to:

- Compare selected model output with analytical solutions, e.g., see [24];
- Compare selected model output with numerical results from other models, e.g., see [25];
- Compare a single parameter or a small fraction of the model output parameters to derive general trends from experiments and modeling, e.g., see [11,20,23].

This study provides an approach to limit the degree of freedom in the input space of RT models for cement-based materials using the results of several experimental investigations at the same time. The proposed approach is based on the monitoring and quantification of changes in the hydrate phases and pore solution at multiple times during early age exposure using (i) thermogravimetric analysis (TGA) to quantify changes of crucial minerals in the solid phase assemblage (Portlandite and Calcite), (ii) X-ray diffraction (XRD) to identify less dominating crystalline minerals in the solid phase assemblage, and (iii) inductively coupled plasma (ICP-OES) measurements of the exposure solution to monitor the change in ionic concentrations of the solution. The approach provides multiple reference data for model testing and validation at different times. Experimental data is used to evaluate predictions of the RT model at an early age with respect to changes in the hydrate phases and pore solution. To demonstrate the applicability of the proposed approach for RT model testing and validation, a case study on the short-term effect of CO_2_ exposure on mineralogical changes of oil-well cement paste is used [26]. Modeling cement carbonation mechanisms is challenging due to the intricate changes in hydrate phase assembly [27,28,29], pore structure [30,31], and pore solution [32]. A combination of experimental results was used to limit the number of possible parameters in the input space of RT models, reducing model uncertainties and is expected to allow for more realistic modeling of long-term exposure with increased accuracy.

## 2. Methodology

### 2.1. Experimental Investigations

A series of short-term carbonation experiments were conducted to test the proposed concept to limit the design space of input parameters for RT models. TGA measurements were used to provide two reference points (Portlandite and Calcite) to follow changes in the hydrate phases over time. In addition, XRD and TGA provided an overview of the crystalline minerals that may be included in the RT model. Finally, ICP-OES measurements provided a reference for changes in elemental concentration in the exposure solution over time, which were utilized to track the interaction between the cement paste and the exposure solution.

#### 2.1.1. Sample Preparation

The carbonation experiments were carried out using class G oil-well cement paste samples. The oxide composition of a typical class G oil-well cement is shown in Table 1, after [33].

Twelve disc-shaped paste samples, 6 mm thick and 85 mm in diameter, were mixed using a high-speed mixer with a w/c ratio of 0.44. The samples were cured for 15 days at 20 °C in a closed container with a wet cloth to maintain 100% relative humidity. After curing, half of the samples were submerged into a 0.5 M NaHCO_3_ solution to study carbonation under saturated conditions. The remaining half of the samples was stored at 20 °C and 100% relative humidity as a reference. The mass ratio between the paste samples and the NaHCO_3_ solution was approximately 1:40 (i.e., each of the approximately 25 g paste samples was submerged into 1 L of solution in a separate container). During the sampling stage, powdered samples were tested by TGA and XRD without using any hydration stoppage technique, as the samples were tested on the day of sampling.

#### 2.1.2. Thermogravimetric Analyses (TGA)

TGA of paste samples was used to quantify changes of Portlandite and Calcite at multiple times during early age exposure to 0.5 M NaHCO_3_ solution. Powdered paste samples, representing a cross-section of the 6 mm thick disc samples, were used to perform thermogravimetric analyses using a NETZSCH STA 449 F3 Jupiter instrument. Samples of approximately 40 mg powder were heated from 30 to 850 °C at 10 °C/min in an N2 atmosphere. The TGA was carried out on carbonated and reference samples after 1, 2, 6, 20, and 42 days of exposure. One reference repetition of the TGA was carried out, which indicated consistent results.

#### 2.1.3. X-ray Diffraction (XRD)

XRD of paste samples was used to identify less dominating crystalline minerals in the solid phase assemblage at multiple times during early age exposure. Powdered paste samples, without hydration stoppage, were used to perform powder X-ray diffraction measurements using a PANalytical X’Pert Pro instrument, operating at 45 kV and 40 mA, applying Cu Kα radiation with a 2θ X’Celerator detector, and a scanning range of 3–65°2θ. The individual peaks of the diffractograms were identified using the X’Pert HighScore Plus software with the database from the International Centre for Diffraction Data (ICDD).

#### 2.1.4. Inductively Coupled Plasma Optical Emission Spectrometry (ICP-OES)

ICP-OES measurements of the exposure solution were used to monitor the change in ionic concentrations of the exposure solution at multiple times during early age exposure. The elemental composition of the exposure solution was measured by IPC-OES (Varian 720-ES ICP-OES) to track the change in water chemistry after 1, 2, 6, 20, and 42 days of exposure of the cement specimens to a 0.5 M NaHCO_3_ solution.

### 2.2. Numerical Model

The numerical model comprised a reactive transport modeling framework developed for the simulation of cement-based systems, as described in [8,11,34,35]. A brief representation of the model framework is provided in this section covering the schemes utilized in this study, i.e., (i) a mass transport model, (ii) a reaction model, (iii) a microstructure model, and (iv) a hydration model.

#### 2.2.1. Mass Transport

The transport part of the model solves an extended version of the Poisson–Nernst–Planck (PNP) system of equations, which is derived from the electro quasi-static hybrid mixture theory [36,37]. The governing mass transport equation is written as follows, assuming a fully saturated system with a negligible impact of the electro-migration term:

(1) $$\frac{\partial c_{i}^{l}}{\partial t}=\nabla \cdot \left(D_{i}^{l}\nabla c_{i}^{l}\right)+q_{i}^{l},$$

where $c_{i}^{l}$ is the concentration of the ith species, the superscript l denotes the liquid phase, t is time, $D_{i}^{l}$ is the effective diffusion coefficient of the ith species in the liquid phase, and $q_{i}^{l}$ is the mass exchange term for chemical interactions between the ith species.

A finite element approach is used to solve the mass transport problem [38]. The weak form of the governing equations is obtained using the Green–Gauss theorem, while the one-dimensional Galerkin’s method is used to discretize the spatial domain with linear elements. Time discretization is carried out using a single parameter implicit time integration scheme. A modified Newton–Raphson scheme is used to account for the non-linearity in the model. Finally, the transport model considers a boundary condition with a variable ionic concentration for this investigation. The boundary condition simulates a specimen immersed in a fixed amount of NaHCO_3_ solution that is not replaced during the experiment, thus assuming fixed ionic concentrations at eh boundary will not be accurate. Therefore, the ionic concentration at the boundary is updated within the numerical framework, considering a fixed water mass. The ionic consternation information from the output of the previous timestep is used in this process. Transport along the boundary domain is neglected so that a perfectly mixed solution is considered at all times (i.e., concentration gradients within the solution are neglected).

#### 2.2.2. Reaction Model (Chemical Equilibrium)

Within the presented approach, chemical equilibrium is considered for reactions among ionic species in the liquid phase as well as the equilibrium between the ionic constituents in the liquid phase and the solid cement hydrate phases to describe the mass exchange terms. Gaseous species (e.g., CO_2_) are only considered fully dissolved in solution, and liquid–gas phase interactions are neglected. For the solution of the chemical equilibrium, the geochemical code IPHREEQC [39,40] is used together with the Cemdata 18 database [19], which uses supplementary data from the PSI/Nagra PSI database [41].

#### 2.2.3. Microstructure Model

The microstructure of the pore system is described in the transport model through a bundle of tubes model, based on a semi-analytical implementation presented in [42]. The model describes variations of the pore-size distribution and corresponding ionic transport properties due to dissolution and formation of phases, calculated by the chemical equilibrium model, as presented in [43]. To account for the effect of changes in the phase assemblage of cement-based materials on the diffusion coefficient, D^i^, of each ion, a tortuosity factor, T_k_, is introduced, which ranges between zero and one:

(2) $$D_{\mathrm{eff}}^{i}=D^{i}T_{k},$$

where $D_{\mathrm{eff}}^{i}$ is the effective diffusion coefficient of the ith ionic species and T_k_ the tortuosity factor at the time-step k, which is calculated after each determination of the chemical equilibrium, following a sigmoid function, as follows:

(3) $$T_{k}=T_{\min}+\left(1-T_{\min}\right)P_{k}^{\left(\frac{\log\left(\frac{T_{0}-T_{\min}}{1-T_{\min}}\right)}{\log\left(P_{0}\right)}\right)},$$

where T_min_ is a lower limit assigned to the tortuosity factor when the pore volume reaches a near-zero value, T_0_ is a reference tortuosity factor assumed at the beginning of the exposure, P_0_ is the total pore volume at the beginning of the exposure, and P_k_ is the total pore volume at the time-step k. It should be noted that T_k_ approaches one, i.e., being $D_{\mathrm{eff}}^{i}$ the ideal self-diffusion coefficient of the ion in pure water $D^{i}$, for P_k_ = 1, i.e., when there are no solid phases present, whereas T_k_ approaches $T_{\min}\approx 0$, when the pore volume reaches values close to zero.

Note that the concept of tortuosity is often used for different purposes interchangeably in the literature [44,45,46,47]. The tortuosity is often defined as a geometric parameter or as a parameter related to the diffusive, hydraulic, or electrical properties [46]. Conceptually, the tortuosity factor T_k_ is a geometric parameter expressed in this study as a ratio 1/L, i.e., higher L values represent a larger tortuosity of the pore structure and, therefore, slower ionic transport.

#### 2.2.4. Input Parameters

Subsequently, the presented reactive transport model was used to simulate the carbonation of class G oil-well cement exposed to 0.5 M NaHCO_3_ solution. The oxide composition of class G cement, as represented in Table 1, was used in the simulation. The degree of hydration was estimated using the TGA measurements along with Equations (4) and (5) [26]. The estimated degree of hydration for the exposed samples (exposure time 14–42 days) was relatively stable, around 60% see also (Figure 2), which justifies the assumption of a fixed degree of hydration during the exposure period (see Table 1). Additional material and model parameters are given in the Appendix A. The input parameters for the RT model are shown in Table A1, the hydrate minerals included in the thermodynamic database for the chemical equilibrium are listed in Table A2, and the diffusion coefficients of the ionic species included are listed in Table A3.

## 3. Results

### 3.1. Thermogravimetric Analysis (TGA)

**Figure 1:**
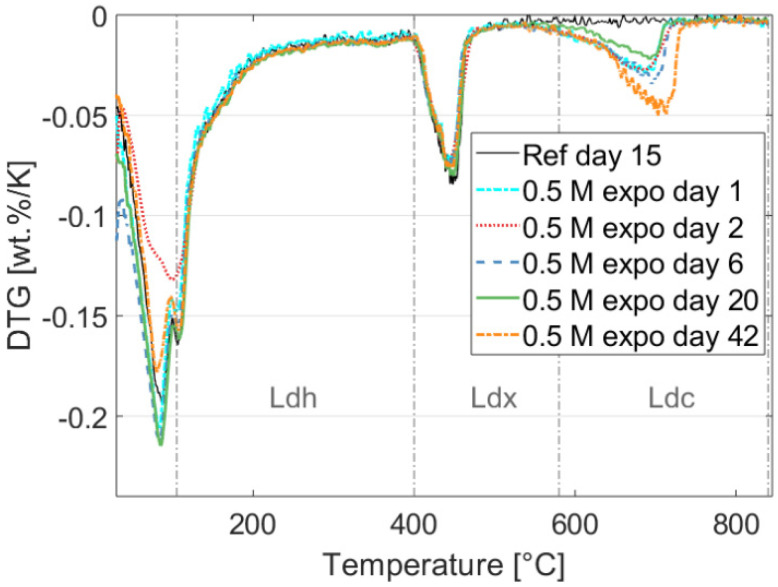
Experimental results showing the effect of exposure on the hydrated phase assemblage of the cement measured by TGA on the reference specimen and during the exposure at 1, 2, 6, 20, and 42 days. The figure shows the temperature ranges for the different decomposition regions of the C-S-H gel (L_dh_), Calcium Hydroxide (L_dx_), and Carbonate phases (L_dc_) (used to estimate the degree of hydration using the method proposed in [49]).

The first derivative of the TGA analysis of a reference sample after 15 days of exposure and samples exposed to 0.5 M NaHCO_3_ for 1, 2, 6, 20, and 42 days are shown in Figure 1. The weight loss in the temperature range up to approximately 400 °C in Figure 1 is typically associated with vaporization of pore water and loosely bound water in C-S-H phases, along with monocarbonate (an AFm phase) in small quantities. The Ldx decomposition region in Figure 1 shows that Portlandite is relatively stable, while the Ldc region indicates that the amount of carbonate increases significantly after the first day of exposure and continues increasing less rapidly afterwards. However, the 20 days exposed sample (green line in Figure 1) shows less carbonate decomposition than the 1-, 2-, and 6-days samples, indicating uncertainties associated with the adopted experimental approach, i.e., the lack of profiling and repetitions of the TGA measurements. More careful testing using depth profiling following [48] should be adopted to improve the accuracy.

**Figure 2:**
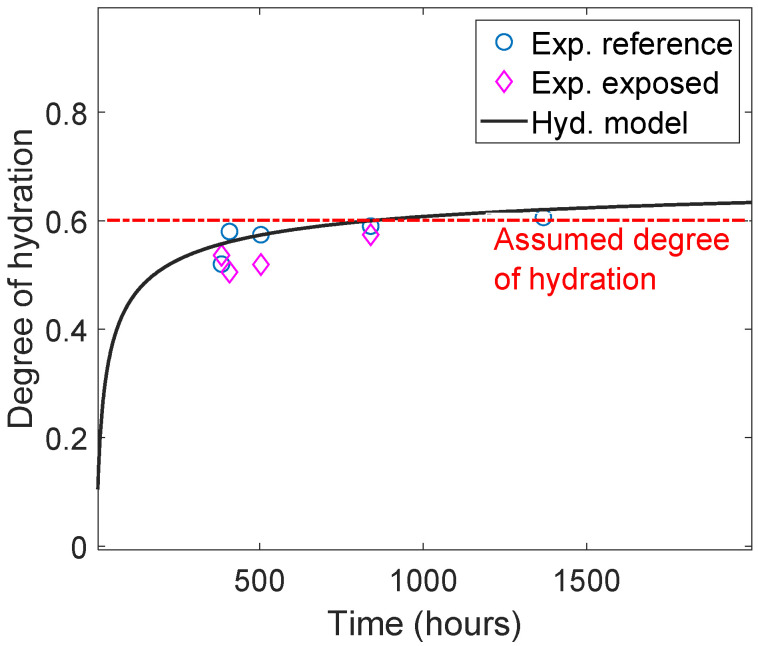
Comparison of experimental results and hydration model to estimate the degree of hydration from TGA measurements using the method proposed in [49]. Note that exposure started after 15 days of curing; hence, the first datum was around 380 h.

Further, TGA measurements were used to get rough estimate of the degree of hydration α using the method proposed in [49], as follows:

(4) $$\alpha =\frac{W_{c}}{0.24},$$

where $W_{c}$ is the chemically bound water defined as

(5) $$W_{c}=L_{\mathrm{dh}}+L_{\mathrm{dx}}+0.41L_{\mathrm{dc}},$$

where $L_{\mathrm{dh}}$, $L_{\mathrm{dx}}$, and $L_{\mathrm{dc}}$ are the mass losses for decomposition regions of the C-S-H gel, calcium hydroxide, and carbonates, respectively. The temperature ranges for the decomposition regions are marked in Figure 2. Note, that we only modeled exposure period, and not the curing time (i.e., from ~400 h to 1500 h in Figure 2). The degree of hydration of the cement can be considered stable during the reactively short exposure time and may be approximated by a constant value of 0.6. A more detailed degree of hydration models should be considered for more accurate representation of the different degree of hydration [15,50].

### 3.2. Phase Assemblage during Exposure (XRD)

**Figure 3:**
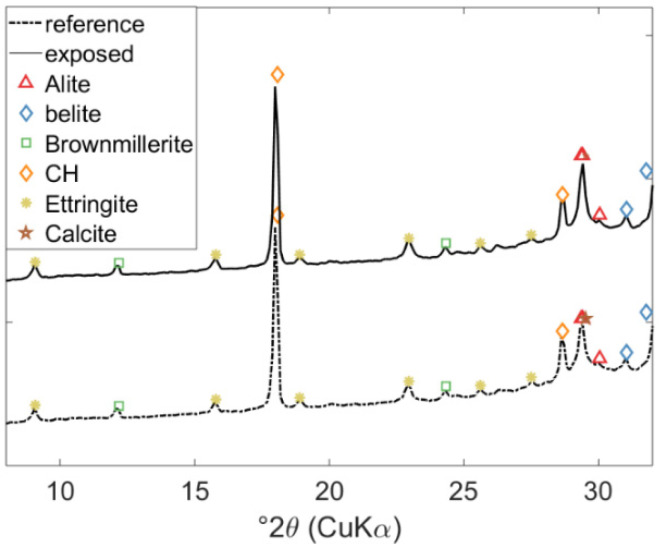
Experimental results showing the effect of exposure on the hydrated phase assemblage of class G oil-well cement measured by XRD, i.e., for reference (unexposed) specimen and after 20 days of exposure.

The measured intensities for the crystalline hydrate phases in the cement paste of a class-G oil-well cement, unexposed and exposed to 0.5 M NaHCO_3_ solution as measured by XRD, are shown in Figure 3. The XRD patterns of the two samples are very similar, indicating the presence of clinker phases (alite, belite, and brownmillerite), ettringite (Aft), Portlandite, and Calcite. The exposed sample has a slightly higher intensity at 29°2θ, indicating slightly more intensity for Calcite. XRD measurements of the remaining samples exposed to 0.5 M NaHCO_3_ solution provided similar information.

### 3.3. Chemical Composition of Exposure Solution (ICP-OES)

Each ion species, such as Ca^2+^ or HCO_3_^−^, will diffuse along concentration gradients for its species towards lower concentrated regions. The pore solution of the cement paste is at the same time in exchange with the exposure solution and near equilibrium with the hydrate phase assemblage. Hence, ionic species that are less concentrated in the exposure solution than in the pore solution will diffuse out of the hardened cement matrix and may lead to the dissolution of a hydrate phase and are, thus, leached (e.g., Ca^2+^). The opposite mechanism is identified as ingress (e.g., HCO_3_^−^). Repeated measurements of the elemental composition, representing the ionic species of the exposure solution over time, provide, therefore, an indication of changes in the pore solution composition.

**Figure 4:**
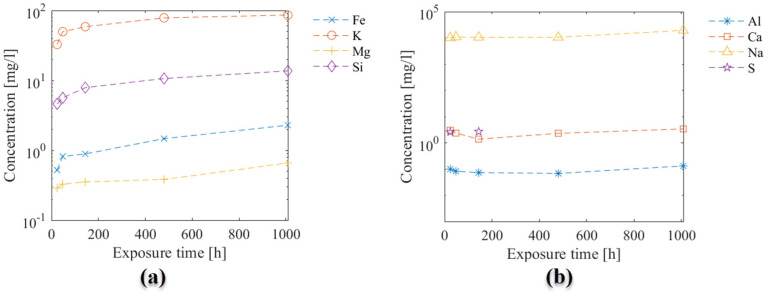
Elemental composition of the exposure solution over time, measured by ICPOES. (**a**) Fe, K, Mg, and Si; and (**b**) Al, Ca, Na, and S, on a different y-scale. Note that Sulphur data are incomplete, and the Al data are around the detection limit.

Results of ICP-OES measurements illustrating the elemental composition in the exposure solution over time are given in Figure 4. The change in concentration over time varied for the different elements: a rapid increase in concentration at the beginning of exposure (following a logarithmic trend) was observed for Fe, K, Mg, and Si (Figure 4a), while a less clear variation over time was observed for Na, Ca and Al (Figure 4b). The data for Sulphur were incomplete, and only values for the first 180 h were available. The concentration of Al was close to the detection limit, and the concertation of Na was very high in the solution from the 0.5 M NaHCO_3_ solution.

### 3.4. Model Results

**Figure 5:**
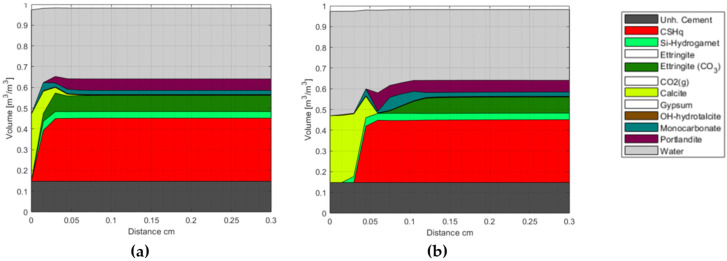
Phase assemblage after (**a**) 1 day and (**b**) 42 days of exposure to 0.5 M NaHCO_3_ solution. The unhydrated cement represents the amount of unreacted cement at the time of hydration. The white blocks in the phase assembly legend indicate phases included in the chemical model, but not present at the displayed times.

Selected modeling results of class G oil-well cement carbonation are shown in Figure 5, illustrating a volume representation of the cement phase assemblage after 1 and 42 days of exposure. The end members of solid solutions listed in Table A2 are grouped in Figure 5 for simplicity. The change in total porosity is represented by the change in water volume, as the system is assumed to be fully saturated at all times.

The presented results show that the carbonation front replaces the majority of the hydrate phases with Calcite, starting with Portlandite, followed by CSH, AFt, and AFm phases, respectively. Furthermore, numerical results indicate that the model can reproduce general trends reported in, e.g., [32,51] concerning the existence of a carbonated zone (~outer 0.01 mm in Figure 5a, and outer 0.03 cm in Figure 5b), a carbonation front (~at 0.03 mm in Figure 5a, and 0.05 cm in Figure 5b), a dissolution front, and an unaffected zone (from ~0.06 cm after 1 day, Figure 5a, and from ~0.12 cm after 42 days, Figure 5b). More detailed analyses of our model representation of these carbonation mechanisms were addressed in [8,11].

### 3.5. Comparison of Model and Experimental Results

**Figure 6:**
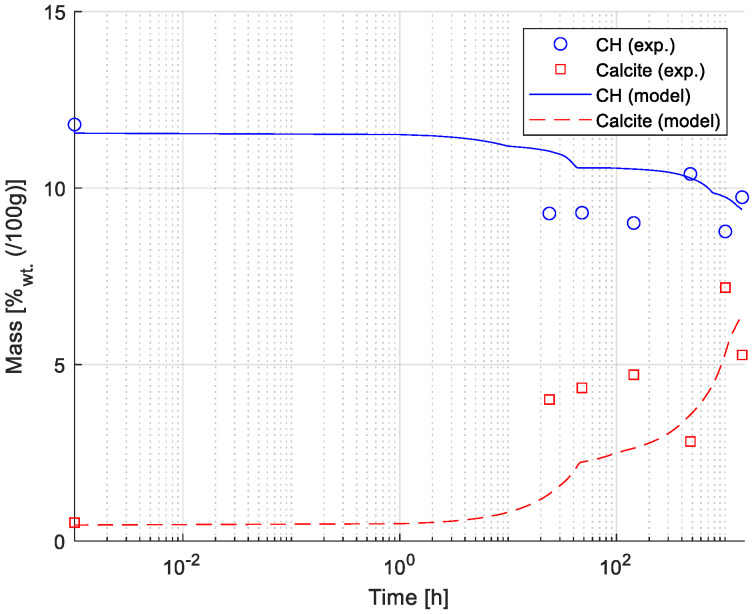
Comparison between model and experimental results for Portlandite (CH) and Calcite. Results presented correspond to the mass fraction (in wt.% of total mass) of Portlandite and Calcite (shown in linear scale) during the exposure time (shown in logarithmic scale). Experimental data is derived from TGA measurements (see also Figure 1).

Figure 6 shows a comparison between numerical and experimental results (estimated employing TGA measurements) for the development of the mass weight percentage of Portlandite and Calcite over time. The mass change profile of Calcite and Portlandite was estimated from the DTG curve (see also Figure 1) using the tangential method [45]. The results show a good agreement between numerically and experimentally determined mass fractions. The carbonation of the paste samples due to exposure to 0.5 M NaHCO_3_ solution drives the dissolution of Portlandite and formation of Calcite, as illustrated in Figure 6.

**Figure 7:**
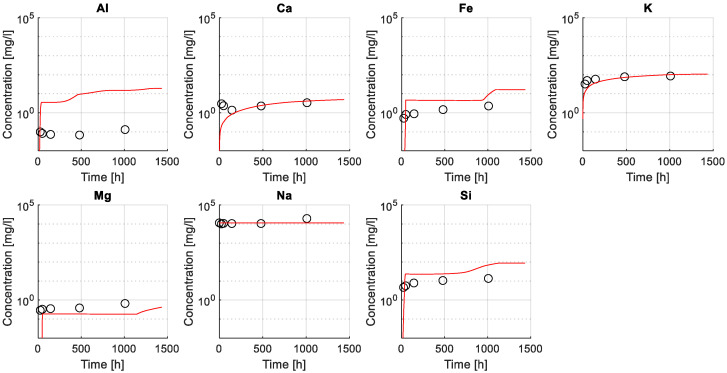
Comparison between numerical and experimental results (ICP-OES) of elemental concentration for selected ions in the exposure solution. Modeled results (lines) and experimental values (markers) are presented in mg/L in a logarithmic scale along the experiment time (presented in a linear scale).

Figure 7 illustrates a comparison between experimental (utilizing ICP-OES, see also Section 2.1.4) and numerical results of the elemental composition of the exposure solution. A good agreement is found between modeled and experimental data, particularly for dissolved elements Ca, K, Si, S, and Mg, while the agreement for the predicted and measured Al concentration in the exposure solution is worse.

Results indicate that the model was able to capture leaching of elements from the sample as shown by an increase in, e.g., Ca and K over time, attributed to the dissolution of less stable hydrated phases in the given exposure condition such as Portlandite or in the case of potassium that is typically not incorporated into hydrate phases. For this reason, the modeled ingress of sodium, due to the high concentration in the exposure solution, is neither captured in the modeled or measured exposure solution under the given conditions. The variation of Fe, Si, and Mg showed a less steep increase, as expected in more stable phases, such as C-S-H, hydrotalcite or siliceous hydrogarnet. The poor fit of the Al concentration in the exposure solution was attributed to limitations in the chemical model to fully capture the stability of aluminum-bearing phases such as AFm and AFt phases.

## 4. Discussion

A quantitative comparison of results presented in Figure 6 showed that the model could reproduce the change in mass for Portlandite and Calcite over time. In addition to Portlandite and Calcite, XRD analyses detected the presence of clinker phases (i.e., alite, belite, and brownmillerite, indicating incomplete hydration of the cement) and ettringite, which is in good agreement with the modeled hydrate phase assembly (see Figure 5). Note that AFm phases have a variable composition and thus may be underrepresented in the experimental part although predicted in the reaction model. Finally, the predicted elemental composition of the exposure solution throughout the exposure time is generally in good agreement with measured values (i.e., using ICP-OES), as illustrated in Figure 7.

**Figure 8:**
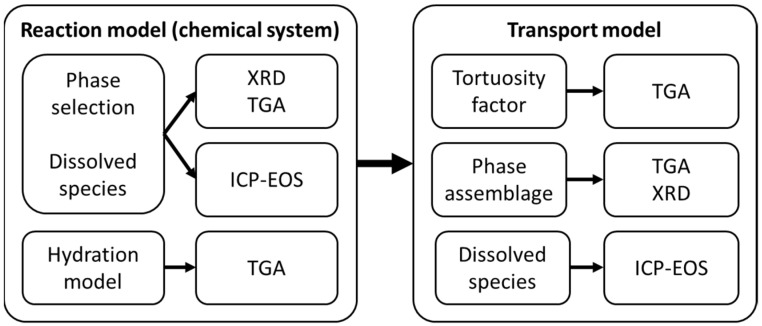
Model validation process, showing the validation of the reaction model (i.e., the chemical system) and the transport model (combined transport and chemistry).

This study aimed to introduce and describe a robust model calibration and validation process that can reduce the degree of freedom in the input space of reactive transport modeling. The proposed process is summarized in Figure 8 and comprises two steps: first, validation of the reaction model, and second, the validation of the transport model. The validation process utilizes collected experimental data describing the phase assemblage (XRD and TGA) and ionic composition of the exposure solution (ICP-EOS), as shown in Figure 8.

### 4.1. Calibration of the Reaction Model

**Figure 9:**
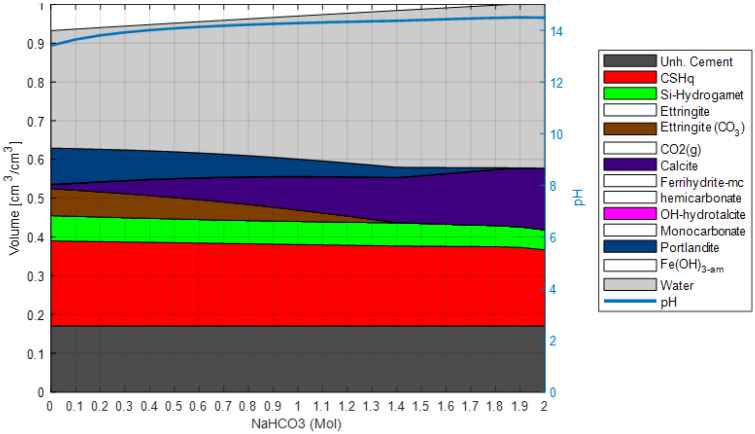
Validation of reaction model. Phase assemblage with increasing concentration of NaHCO_3_ in exposure solution. The unhydrated cement represents the amount of unreacted cement at the time of hydration. The white blocks in the phase assembly legend indicate phases included in the chemical model, but not present at the displayed concentrations of NaHCO_3_ in exposure solution.

The first step in the model validation process is to establish a chemical system coherent with the selected chemical database (Cemdata18 in this case) and the collected experimental data. At this stage, the objective is to identify phases and ionic species that have a role in the chemical system while converging to the most straightforward chemical system possible. This process limits the degree of uncertainty in the model, provides better numerical stability, and reduces computational time. Transport processes are neglected at this stage, and the calibration and validation process of the reaction model comprises the following steps:

1. An initial model of the binder system is set based on the TGA and XRD experimental data. In addition, XRD measurements are used to confirm the phase assemblage of the unexposed hydrated system;
2. The degree of hydration is estimated based on the TGA experimental data (see also Figure 2);
3. The system is simulated with a stepwise increase of NaHCO_3_ in solution (see Figure 9) to validate the considered phase assemblage within the expected modeled chemical systems.

Finally, the robustness of the selected phase assemblage and sensitivity to variation in the oxide composition is checked by evaluating the saturation index of other phases not included in the model (see Table 2). Phases with a negative saturation index are not expected to form and may be neglected in the model, thus simplifying the chemical system. This step in the process is used to confirm that the uncertainties of the initial model assumptions do not significantly impact the model results. After completing this process, the reaction model is expected to represent the studied system and exposure condition and is not modified further during the validation process. Thus, the next step does comprise the calibration of transport parameters solely. This approach can be generalized to cover other exposure conditions by repeating step 3 using the relevant chemical species.

### 4.2. Validation of the Transport Model

**Figure 10:**
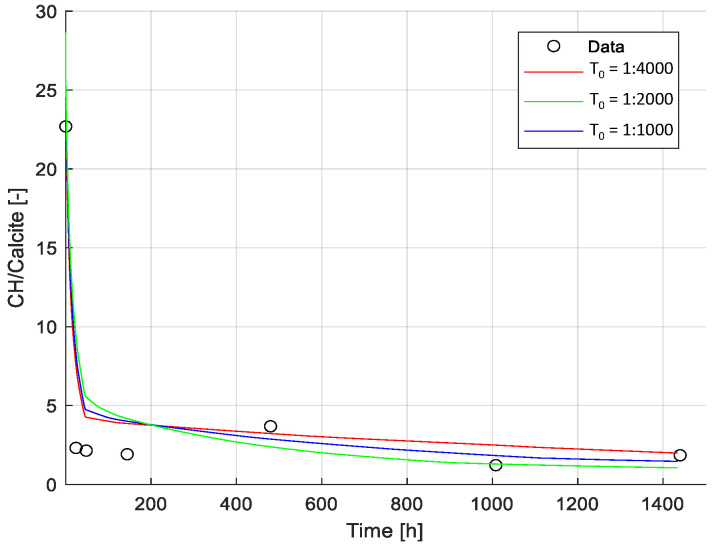
Calibration of tortuosity factor. Comparison between model results (for different tortuosity factors) and experimental data (i.e., Portlandite-to-Calcite ratio). Results presented correspond to the ratio of Portlandite/Calcite during the exposure time (shown in linear scale). Experimental data was derived from TGA measurements. Modeled results are shown for three values of the initial tortuosity factor (T_0_): T_0_ = 1:1000, T_0_ = 1:2000, T_0_ = 1:4000.

It is well known and documented that various exposure scenarios, such as leaching, carbonation, etc., lead to phase changes and consequently changes in the pore volume (see references in [8]. Changes in the pore volume must also affect the transport of various species in the medium; however, this has been studied to a far less extent and even less so incorporated in modeling approaches. One of the first approaches is presented in [8]. However, experimental validation is rather troublesome as a direct observation of changes in the pore volume and mass transport is challenging or even impossible to observe at the nano level (when we talk about pores in the C-S-H phase). Thus, only indirect observations are possible and can be used for testing and validation of modeling approaches. Changes in the phase assemblage, particularly the Portlandite-to-Calcite ratio (CH/Cal.) measured with the TGA, were used as the indirect experimental reference in this study (see Figure 10).

Due to the inclusion of a microstructure model in our framework [8], the only parameter of the transport model adjusted in this example was the tortuosity factor at the initial time of exposure (T0 as described in Section 2.2). However, the impact of this parameter is minimized by the change in tortuosity factor over time, estimated based on the change in hydrate phase composition, as detailed in [8]. The limited impact of T0 on the transport of ions is illustrated by comparing the experimental data with modeling results for T0 values between 1:1000 to 1:4000. Changes in the phase assemblage, particularly the Portlandite-to-Calcite ratio (CH/Cal.) measured with the TGA, were used as the experimental reference (see Figure 10).

**Figure 11:**
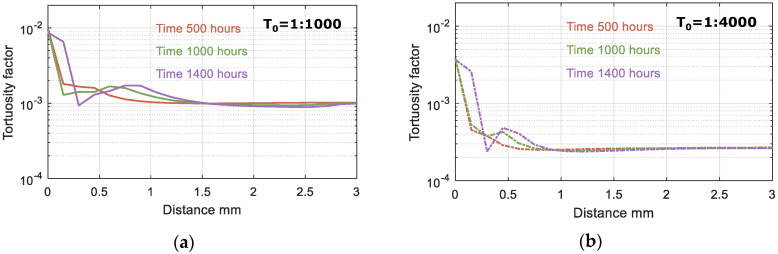
Tortuosity factor after 500, 1000 and 1400 h of exposure. Modeled results are shown for two values of the initial tortuosity factor (T_0_): (**a**) T_0_ = 1:1000 and (**b**) T_0_ = 1:4000.

**Figure 12:**
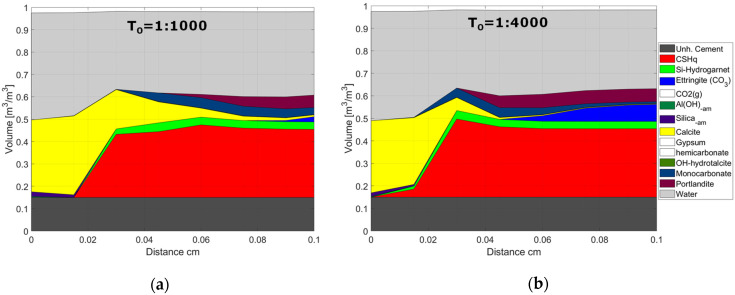
Phase assemblage after 1400 h of exposure. Modeled results are shown for two values of the initial tortuosity factor (T_0_): (**a**) T_0_ = 1:1000 and (**b**) T_0_ = 1:4000. The unhydrated cement represents the amount of unreacted cement at the time of hydration. The white blocks in the phase assembly legend indicate phases included in the chemical model, but not present at the displayed time.

According to the model results, the ingress of HCO_3_, carbonation of Portlandite and C-S-H lead to the precipitation of Calcite, especially in the outer layer of the sample, and an increase in pore volume, resulting in a significant change in tortuosity, as shown in Figure 11 and Figure 12. Based on the change in pore volume, the tortuosity is calculated following Equation (3). Figure 11 illustrates the tortuosity in the outer layer, which increases by one order of magnitude and thus limiting the impact of the initial tortuosity on the overall model results.

This observation is also illustrated in Figure 12, where the phase assemblage after 1400 h of exposure is shown for two values of the initial tortuosity factor. The thickness of the carbonated outer layer and relative change in pore volume are similar for both cases, thus explaining the similar behavior compared to experimental data in Figure 9. It is important to highlight that the modeled changes in pore volume were not validated by experiments in this study and should be investigated further in future studies. However, the overall observed trends are consistent with results reported in the literature, see, e.g., [32,52,53].

In addition, the model predictions of the elemental concentration for selected ions in exposure solution over time (with the three initial tortuosity factors used) were compared to experimental data measured employing ICP-OES (see Figure 4). The change in the chemical composition of the exposure solution is mainly caused by the leaching of ionic species from the pore solution during the exposure time. The leaching of ionic species and variations in the exposure solution over time provides additional data to study transport and chemical reaction processes due to exposure of the sample.

**Figure 13:**
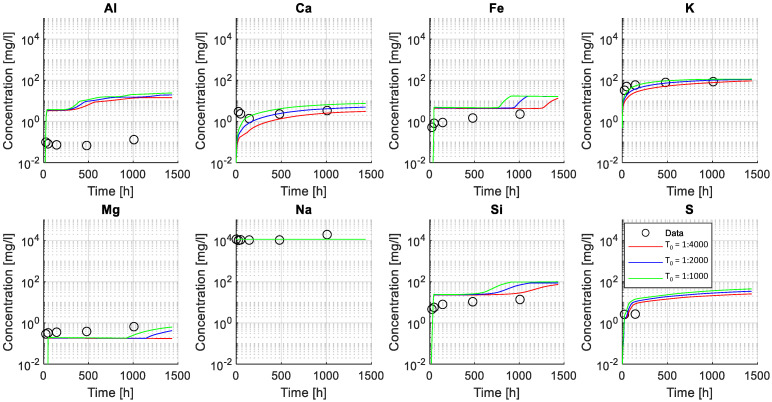
Validation of calibrated tortuosity factor. The figure shows the elemental concentration for selected ions in the exposure solution. Modeled results (lines) and experimental values (markers) are presented in mg/L in a logarithmic scale along the experiment time (presented in a linear scale). Modeled results are shown for three values of the initial tortuosity factor (T_0_): T_0_ = 1:1000, T_0_ = 1:2000, T_0_ = 1:4000.

Figure 13 indicates that the modeled concentration profiles for some of the leached ions (from cement paste into solution) were sensitive to changes of the initial tortuosity (mainly Ca and K). In contrast, the initial tortuosity had less influence on the concentration profile for less soluble elements, i.e., Fe and Si. The concentration of other elements (e.g., Al or Mg) appeared insensitive to changes in the initial tortuosity and were likely governed by the chemical stability of the solid phases and the resulting change in tortuosity. Finally, the large concentration of Na in solution (i.e., from the dissolved NaHCO_3_) hindered its use for calibration and validation as no noticeable change in concentration was observed.

## 5. Conclusions

- In this study, an approach was presented to limit the degree of freedom in the input space of a reactive transport model for cement-based materials;
- The proposed approach utilizes several short-term experimental results, which provide multiple reference data at different times and result in a more robust model validation process;
- Early age carbonation of class G oil well cement exposed to 0.5 M NaHCO_3_ solution was studied as proof of concept;
- Changes in the hydrate phases were evaluated experimentally using TGA and XRD, while the chemistry of the exposure solution was monitored using ICP-OES;
- Comparison between numerical and experimental results indicates that the calibrated model could reproduce changes in hydrate phases and pore solution during early age exposure;
- The process to limit the design space for possible input parameters was carried out in two steps: first, establishing a representative chemical system with a limited number of hydrate phases and ionic species and second, calibrating the transport processes by modifying the impact of the geometric tortuosity on the ionic transport (i.e., through the initial tortuosity factor);
- To establish a representative chemical system, experimental data were used in combination with the Cemdata18 chemical database to identify the dominant hydrate phases and ionic species in the system:
  ○ In particular, TGA experimental data were used to estimate the degree of hydration and provide a reference for the change in mass weight of portlandite and calcite phases over time;
  ○ In addition, XRD analysis detected the presence of clinker phases (i.e., alite, belite, and brownmillerite) and ettringite, which was utilized to describe the initial unexposed hydrated system;
  ○ Repeated XRD measurements over time intervals did not provide additional information; thus, one set of XRD measurements was found to be sufficient when combined with TGA measurements over time for this study.
- The robustness of the calibrated chemical model was tested by simulating a stepwise increase of NaHCO_3_ and varying the initial oxide composition. This process resulted in a representative chemical system with a reduced number of parameters, which limited the degree of uncertainty in the chemical input space of the model, improved numerical stability, and reduced computational time;
- Implementing a microstructure model minimizes the need for a fitting parameter for the transport processes. The microstructure model updates the geometric tortuosity based on changes in the solid phase assemblage;
- The transport processes in the system were tested by modifying the initial tortuosity. The change in Portlandite-to-Calcite ratio measured by TGA and element concentration in the exposure solution measured employing ICP-OES are used as indirect references. Numerical results indicate that the change in the Portlandite-to-Calcite ratio and the leaching of most elements were insensitive to variations in the initial tortuosity factor. The low impact is attributed to the employment of our microstructure model to update the tortuosity based on changes in the solid phase assemblage;
- The use of a microstructure model contributes to improved robustness of the reactive transport model.

## Figures and Tables

**Table 1:** Oxide composition of the class G oil-well cement, after [33] and assumed degree of hydration, DoH.

	CaO	SiO_2_	Al_2_O_3_	Fe_2_O_3_	SO_3_	MgO	K_2_O	Na_2_O
**Mass %**	64.16	21.64	3.89	5.23	2.28	0.79	0.41	0.1
**DoH %**	60	60	60	60	60	60	60	60

**Table 2:** Phase stability. Saturation index of the phases excluded in the system for three scenarios: the original oxide composition (original), an oxide composition with a lower C_3_S content (C_3_S = 48% wt.), and an oxide composition with a higher C_3_S content (C_3_S = 58% wt.). Phases with a negative saturation index are not expected to form.

	Original	C3S = 48%wt.	C3S = 58%wt.
**AFt-phases**			
Tricarboaluminate	−0.55	−0.54	−0.55
**AFm-phases**			
Hemicarbonate	−0.74	−0.74	−0.74
Monosulphate14	−1.39	−1.39	−1.39
Straetlingite	−2.95	−2.94	−2.95
Fe-hemicarbonate	−5.60	−5.60	−5.60
Fe-monocarbonate	−3.25	−3.25	−3.25
**Hydroxides**			
Al(OH)_3(am)_	−4.07	−4.07	−4.07
Al(OH)_3(mic)_	−3.21	−3.21	−3.21
FeOOH_(mic)_	−1.29	−1.29	−1.29
SiO_2 (am)_	−6.25	−6.25	−6.25

## Appendix A

**Table A1:** Reactive Mass Transport (RMT) model input parameters.

Parameter	Value	Unit
**General parameters**		
Total distance	3	mm
Total time	90	days
Temperature	20	°C
Pressure	1	bar
**Transport model**		
Initial tortuosity (T_0_)	1:2000	-
Shape factor (τ)	1	-
Initial porosity (ω0)	0.4	-

**Table A2:** Thermodynamic database for the chemical equilibrium model, from [16].

Equilibrium Phases	
**Portlandite**	OH-hydrotalcite
Calcite	Monocarbonate
Gypsum	CO_2_ (g)
**Solid solution phases**	
**C-S-H**	
CSHQ-JenD	CSHQ-JenH
CSHQ-TobD	CSHQ-TobH
NaSiOH	KSiOH
**Ettringite**	
ettringite	ettringite30
**SO4_CO3_AFt**	
tricarboalu03	ettringite03_ss
**Si-Hydrogarnet**	
C3AFS0.84H4.32	C3FS0.84H4.32

**Table A3:** Diffusion coefficients (Di ×10^−9^) of the ionic species included in the numerical example, after [31].

OH^−^	5.30	CaCO_3_	0.45	Mg(OH)^+^	0.71	NaCO_3_^−^	0.71
H^+^	9.31	CaSiO_3_	0.71	Mg^+2^	0.71	NaHCO_3_	0.71
AlO_2_^−^	0.71	Ca(HCO_3_)^+^	0.47	Mg(SO_4_)	0.71	SO_4_^−2^	1.07
AlO_2_H	1.04	Ca(HSiO_3_)^+^	0.71	Mg(CO_3_)	0.71	HSO_4_^−^	1.39
AlSiO_5_^−3^	0.71	FeO_2_^−^	0.71	MgSiO_3_	0.71	CO_2_	2.26
AlO^+^	0.71	FeO_2_H	0.71	Mg(HCO_3_)^+^	0.71	CO_3_^−2^	0.96
Al(OH)^+2^	1.04	FeO^+^	0.71	Mg(HSiO_3_)^+^	0.71	HCO_3_^−^	1.18
Ca(OH)^+^	0.16	K^+^	1.96	NaOH	1.33		
Ca^+2^	0.16	KOH	1.96	Na^+^	0.03		
CaSO_4_	0.47	KSO_4_^−^	0.75	Na(SO_4_)^−^	0.62		
